# Influence of information concerning a computerized anesthesia system on dental anxiety: a randomized controlled clinical trial

**DOI:** 10.4317/medoral.23315

**Published:** 2020-02-10

**Authors:** Alejandro Rizzo-Lorenzo, Alba Sánchez-Torres, Carlos Noguera-Mutlló, Irene Pérez-Beltrán, Rui Figueiredo, Eduard Valmaseda-Castellón

**Affiliations:** 1 DDS. Fellow of the Master of Oral Surgery and Implantology, School of Medicine and Health Sciences, University of Barcelona, Barcelona, Spain; 2 DDS, MS. Associate Professor of Oral Surgery. Master’s Degree Program in Oral Surgery and Implantology, School of Medicine and Health Sciences, University of Barcelona. Researcher of the IDIBELL institute, Barcelona, Spain; 3 DDS, MS, PhD. Master of Oral Surgery and Implantology. Associate Professor of Oral Surgery, School of Medicine and Health Sciences, University of Barcelona, Barcelona. Researcher at the IDIBELL Institute. Barcelona, Spain; 4 DDS, MS, PhD, EBOS. Professor of Oral Surgery, Director of the Master of Oral Surgery and Implantology, School of Medicine and Health Sciences, University of Barcelona. Researcher at the IDIBELL Institute. Barcelona, Spain

## Abstract

**Background:**

A single-blinded randomized controlled trial among patients requiring an upper third molar extraction was performed to evaluate the anxiety degree after receiving information or not about the functioning of The Wand system. Secondarily, perceived pain and the need of re-anesthesia were assessed.

**Material and Methods:**

Patients were randomly assigned to the experimental group (detailed explanation about The Wand) or control group (no specific information). Local anesthesia with The Wand consisted in a supraperiosteal infiltrative technique injection 1.6 mL at the buccal and 0.2 mL at the palatal side. Distinct questionnaires for assessing dental anxiety and 100-mm visual analog scales to assess pain were delivered. Demographic data, radiological parameters, operative time and type of intervention were also registered. A descriptive bivariate analysis by non-parametric tests to detect differences in anxiety, pain and re-anesthesia was performed by SPSS 22.0 (SPPS Inc. Chicago, USA).

**Results:**

A total of 85 patients were assessed for eligibility but 17 participants were lost due to the cancellation of the visit for the surgical intervention. Finally, sixty-eight patients were included (34 participants in each group), 47 women (69.1%) and 21 men (30.9%), with an average age of 28.8 (± 9.3) years.

**Conclusions:**

Patients that received a detailed explanation of The Wand did not have a significant reduction of the anxiety degree and perceived pain during the anesthetic act compared to patients that received no information. The need of re-anesthesia was not related to the anxiety level but was significantly related to increasing operative time.

** Key words:**Dental anxiety, pain, local anesthesia, third molar, computerized anesthesia.

## Introduction

Anxiety and pain during dental procedures are well described in the literature (1-4). Despite current improvements on materials and techniques, dental anesthesia is usually one of the most feared parts of dental procedures. This could difficult our maneuvers and create discomfort to patients (1,5). Pain during anesthesia can be generated by the puncture or by the fluid release (1-3,5-8) and worsened by anxiety and fear from patients (1,7,8). Injecting slowly at a low pressure seems to reduce pain and increase comfort during dental anesthesia (2,6).

The research into this field aims to develop less invasive and painful systems compared to conventional anesthesia (4,9,10). The Wand (Milestone Scientific, Deerfield, IL) was designed to decrease pain and anxiety produced by the traditional anesthetic syringes. It consists in a computerized controlled injection activated by a footswitch pedal (3,5,7-11). The hand piece is similar to a pen, which constitutes an advantage as it does not remind a conventional syringe (3,7). The device maintains the anesthetic liquid flow at a constant volume and pressure, independently from the tissue resistance (3-5,7-11), and it can be used at a slow or high speed (4-6,10). Presumably, it relieves few drops that precede the needle puncture, thus, creating a virtually imperceptible injection (3,5,9,10). This system delivers mild beeps during the procedure. However, some studies speculate that it could produce some patients to be more nervous because this is a new and unknown system for them compared to the traditional syringe (3,10).

The palatal injection is usually painful (3,5,10,11). As the speed of liquid flow seems to be the main cause (5), the low speed injection is recommended in areas of dense tissue such as palatal mucosa (4,11). A study published by Shah *et al*. (5) compared the perceived pain of palatal injections by The Wand or by traditional syringe and obtained better results with The Wand. Interestingly, some studies made in children did not find relevant differences in terms of pain for highly anxious individuals (4,9). As anxiety may reduce the threshold of pain and patients waiting for a surgical procedure experience anxiety, the misunderstanding or the lack of information about the procedures could even worsen their feelings and pain response. Into this field, there is a lack of studies addressing the benefits of giving information about the anesthetic technique.

The aim of this study was to evaluate the anxiety degree after receiving information or not about the mechanism of The Wand system, previous to an upper third molar extraction. The secondary aim was to assess the perceived pain and need of re-anesthesia.

The hypothesis was that previous explanation would reduce the anxiety levels and perceived pain after the anesthetic act. Furthermore, very anxious patients would need more re-anesthesia during the surgical procedure than less anxious individuals.

## Material and Methods

This study has been carried out according to the CONSORT (Consolidated Standards of Reporting Trials) guidelines (12). All patients signed an informed consent, and the study protocol was approved by the ethical review board of the Dental Hospital of the University of Barcelona (Protocol number 11/2016). The Declaration of Helsinki guidelines were followed throughout the trial.

- Experimental design

A single-blinded randomized controlled trial was performed among patients requiring an upper third molar extraction treated by postgraduate students of the Master of Oral Surgery and Orofacial Implantology at the dental hospital of the University of Barcelona.

- Participants

Inclusion criteria were patients from 18 to 45 years old, coming for an upper third molar extraction (either erupted, partially or totally included) in absence of symptomatology, willing to participate in the study and signed the informed consent for treatment.

On the contrary, exclusion criteria were dental professionals or students because of the possible knowledge of the anesthetic system, patients with systemic diseases (≥ASA III), suffering from allergy or intolerance to the local anesthesia administered, patients who needed other tooth extraction in the same surgical act, surgical interventions lasting more than 60 minutes, active infection in maxillofacial area, concomitant treatment with systemic antibiotics and/or analgesics and abnormal hemodynamic parameters (heart rate <50 o >110 beats per minute, systolic blood pressure <70mmHg o >150mmHg and diastolic blood pressure <50mmHg o >100mmHg).

- Study intervention

At the 1st visit, the patients answered the STAI-T (State-Trait Anxiety Inventory) questionnaire to identify their anxiety trait. Then, in a 2nd visit when the patients came to perform the surgical procedure, they were randomly assigned to the experimental group (detailed explanation about the functioning of The Wand system) or control group (no specific information about the system) by means of a computer generated random sequence through www.randomization.com web page.

The explanation for experimental group was verbal and standardized: “The Wand is a local anesthesia system whose main objective is to reduce pain and anxiety during the anesthetic act. It has a pen-shaped design producing less visual impact, thus reducing patient anxiety. Moreover, this device releases anesthesia regardless of tissue resistance, at a low pressure and a constant flow, which produces less pain during puncture and injection. In your case, the anesthetized area will be the buccal gingiva and the palate associated to the third molar that is going to be extracted”. No explanation was given to participants belonging to the control group.

- Registered variables

Anxiety degree measured by distinct questionnaires (Interval Scale of Anxiety Response (ISAR), Modified Dental Anxiety Scale (MDAS), Dental Fear Survey (DFS) and State-Trait Anxiety Inventory (STAI)) was considered the primary outcome variable. Perceived pain measured by 100 mm visual analog scales (VAS) in 4 different times (palatal and buccal puncture and anesthetic infiltration) and need of re-anesthesia during the surgical procedure were considered secondary outcome variables. Demographic data (age and gender), radiological parameters (Pell & Gregory (13), Winter (14) and Parant (15) classifications), operative time (measured in minutes; from incision to the last suture knot) and type of intervention (with or without ostectomy and/or odontosection) were also registered.

The ISAR questionnaire consists in drawing a horizontal stroke into a 90 mm vertical VAS to set the anxiety level. The MDAS is a modification of Corah’s Dental Anxiety Scale that contains 5 questions measuring anxiety in distinct stages of a dental treatment. The maximum punctuation is 25 and patients with a mark ≥ 19 should be considered as very anxious. DFS is specifically designed to measure dental anxiety. It has 20 questions and the total punctuation varies from 20 (no fear) to 100 (terrified). The cut-off point that differentiates patients with or without dental anxiety is considered to be at 63. The STAI questionnaire consists in 40 questions divided in two groups; the first evaluates anxiety as a transitory state (anxiety state) and the second, as a latent feature or trait (anxiety trait). Anxiety trait (STAI-T) identifies relatively sTable individuals who have a tendency to perceive some situations as a threat. Thus, patients answered this questionnaire at the 1st visit. On the other hand, state anxiety (STAI-S) is considered to be a transient emotional state related to subjective feelings, apprehension and hyperactivity originating from autonomous nervous system.

- Surgical technique

Three blinded and previously calibrated researchers (AST, CNM, IPB) performed the local anesthesia with The Wand system by a supraperiosteal infiltrative technique injection 1.6 mL at the buccal and 0.2 mL at the palatal side, using a short dental needle of 25 mm and 30 G (Artinibsa; Inibsa, Lliçà de Vall, Spain) and one cartridge of 4% articaine with 1:100.000 epinephrine (Artinibsa; Inibsa, Lliçà de Vall, Spain). After concluding the anesthetic act, one investigator (ARL) delivered the questionnaires (ISAR, MDAS, DFS, STAI-S) and the four VAS scales were administered at the same time.

The surgeries were performed by the Master fellows with similar experience and the same technique. The surgical technique employed for partially or totally impacted third molars consisted in raising a full-thickness flap to perform bone removal (if needed) with a hand piece at high speed (40.000 rpm) and a tungsten carbide round bur with constant sterile saline solution irrigation. The wound was closed with 3/0 silk interrupted suture.

- Sample size calculation and statistical analysis

A sample size calculation with an α – error of 0.05, a power of 0.9 with and a size effect d=0.8 was performed by G* Power software version 3.1.9.2 (Universität Kiel, Germany). Size effect (d) was calculated from a previous sample of patients anesthetized with The Wand® system. The total sample was 68 patients divided into two groups of 34 individuals (experimental and control group) each one.

A descriptive and bivariate analysis was performed by SPSS 22.0 (SPPS Inc. Chicago, USA). Normality was assessed by means of Saphiro-Wilk test and non-parametric tests were used in case of absence of a normal distribution to detect differences for anxiety, pain and need of re-anesthesia between the experimental and control groups. The results of MDAS and DFS questionnaires were analyzed as dichotomic variables. The level of significance was set at *p* < 0.05.

## Results

**Figure 1:**
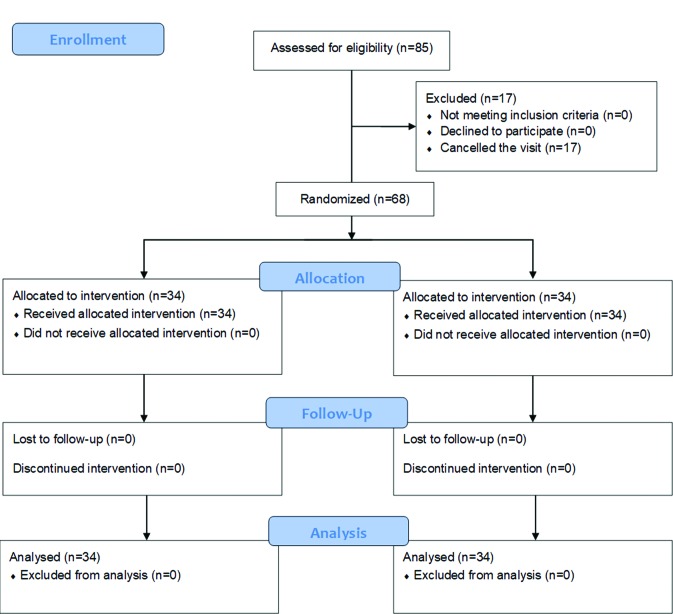
Flow chart of the participants along the study according to the CONSORT guidelines.

A total of 85 patients were assessed for eligibility at the 1st visit although 17 participants were lost due to the cancellation of the visit for the surgical intervention. Fig. 1 shows the flow chart of the participants along the study according to the CONSORT guidelines (12). Finally, this study comprised 68 patients, 47 women (69.1%) and 21 men (30.9%), with an average age of 28.8 (± 9.3) years (ranging from 18 to 67). A total of 30 (44.1%) right and 38 (55.9%) left upper third molars were extracted in a mean operating time of 19.2 (± 11.7) minutes. According to Parant classification (15), 55 third molars were extracted conventionally (Parant type 1), while 13 needed ostectomy to be extracted (Parant type 2). Table 1 shows the Pell & Gregory (13) and Winter (14) classification distributions.

**Table 1:**
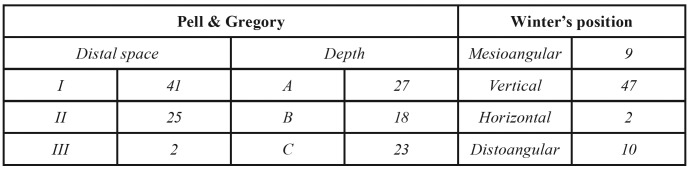
Radiological variables. Pell & Gregory and Winter’s position distributions.

The results of the anxiety questionnaires for the overall sample and for each study group are highlighted in Table 2. Most scores were low and thus, there were few individuals being highly anxious. As these variables did not have a normal distribution, non-parametric tests were used (U-Mann Whitney for continuous and Fisher test for dichotomic variables). The previous explanation did not influence the anxiety level measured by questionnaires as there was no difference between groups.

Table 3 shows no differences for VAS scores for pain during vestibular and palatal puncture and infiltration between groups.

**Table 2:**
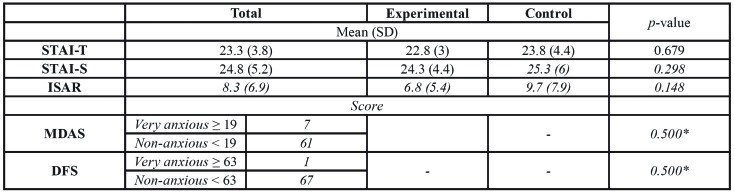
Primary outcome variable. Anxiety scores for the overall sample and for each study group according to different questionnaires. Calculated by U-Mann Whitney test except for dichotomic variables (Fisher exact test) marked with “*”. SD: standard deviation; STAI-T/STAI-S: State-Trait Anxiety Inventory; ISAR: Interval Scale of Anxiety Response, MDAS: Modified Dental Anxiety Scale; DFS: Dental Fear Survey.

**Table 3:**
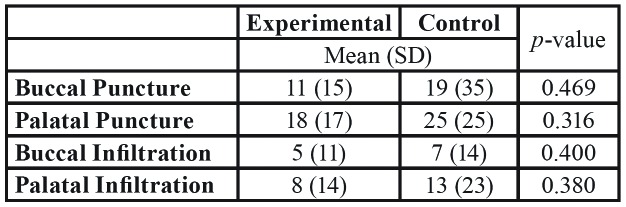
Perceived pain (100-mm VAS score) during buccal and palatal puncture and infiltration, and *p-value*s (U-Mann Whitney test) between groups.

**Figure 2:**
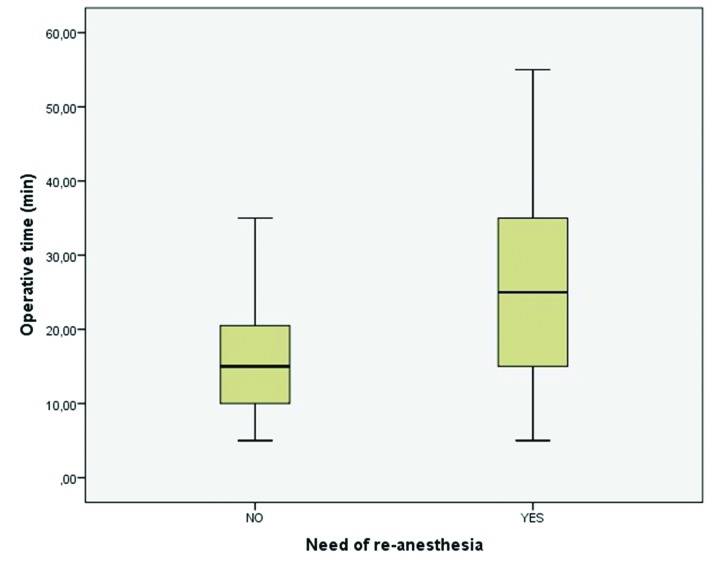
Box-plot rendering the need of re-anesthesia according to operative time (*p*=0.007).

A total of 29 (42.6%) patients had to be re-anesthetized intraoperatively. There was a statistically significant relation with the increasing operative time (*p*=0.007), as the surgical interventions from patients with no need for additional anesthesia lasted for 15.5 (SD=8.8) minutes and the ones that had to be re-anesthetized lasted for 24 (SD=13.3) minutes. Fig. 2 shows two box-plot rendering the distribution of operative time according to the need of re-anesthesia.

No significant differences were found for the need of re-anesthesia depending on the level of anxiety measured by the distinct questionnaires (Table 4) or by previous explanation (*p*=0.806). Moreover, no significant relationship was found neither between re-anesthesia and radiological variables (distal space: *p*=0.245; depth: *p*=0.400; Winter’s position: *p*=0.235) nor with the type of intervention (Parant: *p*=0.059).

**Table 4:**
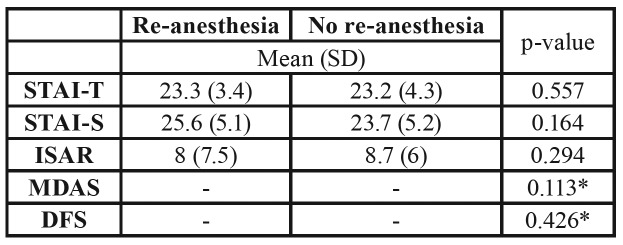
Results of the different anxiety questionnaires in patients that needed intraoperative re-anesthesia versus those who did not. Calculated by U-Mann-Whitney test except for dichotomic variables (Fisher exact test) marked with “*”. SD: standard deviation; STAI-T/STAI-S: State-Trait Anxiety Inventory; ISAR: Interval Scale of Anxiety Response, MDAS: Modified Dental Anxiety Scale; DFS: Dental Fear Survey.

## Discussion

The fear concerning the pain from the anesthetic injection is very frequent (2) and these patients could report greater pain during the anesthetic act compared to patients without fear (16).

We selected the upper third molar removal as a model to our study because it is a very reproducible model of surgical intervention. In particular, palate is one of the most painful areas (17) and this issue could help to really determine the efficacy of the intervention studied, that is, previous explanation of the anesthetic system. This study did not use topical anesthesia in order to reduce potential bias at collecting VAS scores for pain. However, the fact that anxiety and pain are subjective variables, make comparisons with the general population difficult, and this could be one of the limitations of this study. To avoid this, distinct questionnaires were used in order to reduce bias.

This study showed that previous explanation of the anesthetic system does not decrease the level of anxiety, as measured by distinct questionnaires. Therefore, no statistically significant differences between groups were noticed. However, the fact that individuals with a previous upper third molar extraction were also included could constitute a bias when answering the anxiety questionnaires. To date, there are no studies addressing the anxiety depending on the information delivered to the patient about the anesthetic system. Recent research (18) found that the information provided to patients did not changed the overall satisfaction after an impacted third molar extraction. However, the amount of information is a controversial issue as some patients could experience more anxiety due to an excess of that.

Computerized controlled anesthetic delivery systems are able to better control the flow and pressure of the liquid which can reduce pain during local anesthesia infiltration (6,17,19). A recent literature review (19) showed that most studies compared conventional dental anesthesia with computerized controlled anesthesia systems in terms of pain, generally obtaining better results in the maxilla for the computerized one, as measured by visual analogue scales, electric pulp tester, dental anxiety scale, or perceived stress scale. Nevertheless, the sound and beeps emitted by The Wand System, the slowness of the anesthetic act and the fact that to observe an unknown anesthetic device could even provoke more fear that a traditional syringe are some unpleasant issues discussed in some studies (10,19).

A study published by Wang *et al*. (20) was performed to know the individual concerns about dental anxiety on patients previously diagnosed with MDAS questionnaire as anxious individuals. The investigators interviewed the participants to obtain suggestions for improving their anxiety in front of a dental treatment. Interestingly, they all answered that having more information regarding treatment indication and treatment steps could help them to understand the process and to be able to ask questions to the professional and therefore, reduce the perceived anxiety. Especially, they agreed that seeing real images of treatment or dental instruments close to their mouths as an example of the “tell-show-do” technique would exacerbate anxiety. Likewise, a randomized clinical trial performed by Heaton *et al*. (16) studied the ability of a computerized program based on systematic desensitization (CARL) and an informative pamphlet to reduce dental injection fear. The results showed a greater reduction of fear to the anesthetic act for patients that had followed CARL compared to the ones reading the leaflet. Both studies support the hypothesis that information or explanation of a dental procedure could reduce perceived anxiety and even pain, but contradict our results. However, one limitation of the present study is the fact that few very anxious patients were included, as shown by questionnaires in Table 2. Probably, more differences between the experimental (explanation) and control (without explanation) groups would have been found in the presence of more extremely anxious patients.

There are no studies performed in adults with computerized controlled anesthetic delivery systems that assess the need of re-anesthesia during a surgical procedure. Concerning the present study, the need of re-anesthesia (a total of 42.6%) was correlated to increasing operative time but not to the anxiety level nor to the previous explanation. We can assume that it is a high rate for re-anesthesia given the mean operative time. However, the fact that surgical procedures were performed by fellows could overestimate the results as some cases could have been treated as an intraoperative painful sensation in order to detect patients feeling uncomforTable with the exerted pressure during the surgery.

Interestingly, a randomized clinical trial from Patini *et al*. (21) that treated children between 5 and 12 years for contralateral extractions observed four times more need of intraoperative re-anesthesia by using the conventional syringe anesthesia compared to The Wand system, although no information about operative time is available. The use of a computer-controlled delivery device seems to achieve more diffusion of the anesthetic solution than manual syringe (11).

Overall, the use of a computerized anesthesia system seems to be preferred as it can reduce disruptive behavior in children (3) and pain during injection (11,17,22). A prospective cohort study (22) reported that more than a half of the patients included would even pay additionally to be anesthetized by a minimally invasive system, thus confirming their positive experience respect to traditional injection.

To conclude, patients that received a detailed explanation of The Wand system previous to an upper third molar extraction did not have a significant reduction of the anxiety degree and perceived pain during the anesthetic act compared to patients that received no information.

The need of re-anesthesia was not related to the anxiety level but was significantly related to increasing operative time.
